# A System of the Practice of Medicine; from the Latin of Dr. Hoffman, in Two Volumes

**Published:** 1784

**Authors:** 


VIII. *A Syſtem of the Practice of Medicine; from the Latin of Dr.* Hoffman, *in two volumes. By the late* William Lewis, M. B. F. R. S. *author of the New Diſpenſatory, &c. Reviſed and corrected by* Andrew Duncan, M. D. *fellow of the Royal College of Phyſicians, Edinburgh; and member of the Royal Societies of Medicine, of Paris, Copenhagen, Edinburgh, &c.* 8vo. Murray, *London*, 1783. Vol. I. p. 591. Vol. II. p. 584. 12s.

THE works of Hoffman have long been held in high eſteem by medical readers; but from their voluminous ſize, joined to the circumſtance of their being written in the Latin language, they have been leſs extenſively uſeful in this country, than might otherwiſe have been the caſe. The public, therefore, we have no doubt, doubt, will receive with pleaſure this abridged Engliſh tranſlation of his *Medicina rationalis ſyſtematica*, which is confeſſedly the moſt valuable of his writings. It may be objected by ſome readers, and not without reaſon, that the number of remedies which he has recommended in different diſeaſes, is much greater than intelligent practitioners in this country now employ; but at the ſame time, as the learned editor of this abridgment very properly remarks, the fidelity, extent, diſcernment, and accuracy of his obſervations cannot fail to ſtamp a high value on his work in the opinion of every reader, who prefers uſeful facts to fanciful ſpeculation.

The MS. copy of this abridgment was one of thoſe manuſcripts which we formerly mentioned (vol. II. p. 52.) as having been ſold with the reſt of the late Dr. Lewis's library. It was ſoon after put into the hands of Dr. Duncan, who has carefully reviſed and compared it with the original; but the attention he has beſtowed on it, as he informs us in his preface, has ſerved rather to convince him of the fidelity with which it was executed, than to add to the value of the abridgment. He has, however, introduced ſeveral diſeaſes, which Dr. Lewis had probably omitted from their being of little importance, or rarely occurring in this country. By this means every part, section, and chapter of this abridged translation correspond exactly with the same divisions in the folio edition of Hoffman's works published at Geneva in 1761.

